# Post-translational protein lactylation modification in health and diseases: a double-edged sword

**DOI:** 10.1186/s12967-023-04842-9

**Published:** 2024-01-10

**Authors:** Hang Gong, Huang Zhong, Long Cheng, Liang-Ping Li, De-Kui Zhang

**Affiliations:** 1 Department of Gastroenterology, Lanzhou University Second Hospital, Lanzhou, Gansu China; 2 Department of Gastroenterology, Zigong First People’s Hospital, Zigong, Sichuan China; 3 Department of Gastroenterology, Sichuan Academy of Medical Sciences and Sichuan People’s Hospital, Chengdu, Sichuan China

**Keywords:** Lactate, Lactylation, Post-translational modification

## Abstract

As more is learned about lactate, it acts as both a product and a substrate and functions as a shuttle system between different cell populations to provide the energy for sustaining tumor growth and proliferation. Recent discoveries of protein lactylation modification mediated by lactate play an increasingly significant role in human health (e.g., neural and osteogenic differentiation and maturation) and diseases (e.g., tumors, fibrosis and inflammation, etc.). These views are critically significant and first described in detail in this review. Hence, here, we focused on a new target, protein lactylation, which may be a “double-edged sword” of human health and diseases. The main purpose of this review was to describe how protein lactylation acts in multiple physiological and pathological processes and their potential mechanisms through an in-depth summary of preclinical in vitro and in vivo studies. Our work aims to provide new ideas for treating different diseases and accelerate translation from bench to bedside.

## Introduction

Post-translational modifications (PTMs) refer to the chemical modification of a protein after translation and regulate protein activity, localization, and folding, as well as critical interactions between proteins and other biomacromolecules [1, 2]. Many important life activities and the occurrence of diseases have been linked not only to the abundance of proteins but also to various post-translational modifications of proteins [3]. Proteomic modifications are of great significance to reveal the mechanisms of life activities, screen clinical markers of diseases, and identify drug targets through an in-depth study of the changes in protein post-translational modification levels [4]. It has long been believed that lactate is a metabolic waste of glycolysis by cellular life activities under hypoxia, thus prompting the stereotype formation of lactate as a harmful substance [5]. However, biological functions of lactate are being progressively discovered, including intracellular energy supply, signal transduction, modulation of the tumor microenvironment, inflammation regulation, etc., and are also involved in the progression of cancer, inflammatory diseases, and metabolic diseases [6–12].

Several common PTMs, such as acetylation, methylation, ubiquitination, and phosphorylation, have received widespread attention and have been well characterized [13]. Interestingly, a lactate-induced lactylation modification of histone lysine residues was first identified in 2019 by Zhang et al. [14] and was involved in the homeostatic regulation of M1 macrophages under bacterial infections. Protein lactylation proposed as a new PTM not only opens up a new field for the study of proteins but also indicates a novel direction for exploring lactate in cancer, metabolism, immunity, etc. This review elaborates on this topic based on lactate metabolism and the effects of histone or non-histone lactylation on cellular biology. It contributes to further understanding protein lactylation and elucidating the role of lactate in the regulation of cell function. Finally, we explore the possibility of targeting potential targets of lactylation modification for the treatment of various diseases.

## Lactate

### The lactate shuttle

**Fig. 1:**
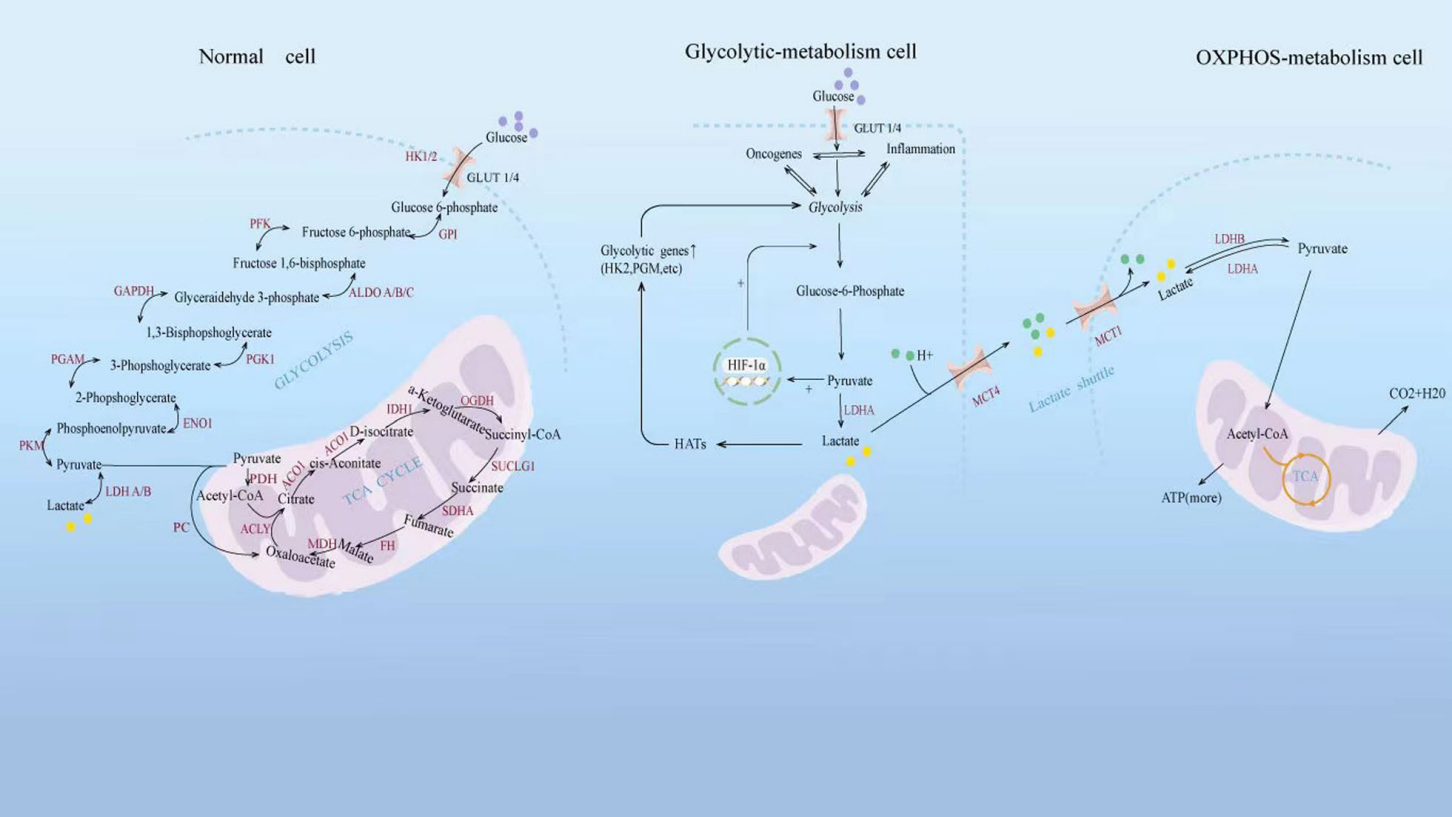
Regulation of lactate metabolism progress in normal, glycolytic and oxidative cells. Glucose metabolism mainly contains glycolysis and the TCA cycle in the mitochondrion. With sufficient oxygen, normal cells produce energy mainly through the TCA cycle. Under stimulation with hypoxia, tumors, and inflammation, glycolytic cells trigger a large uptake of glucose and participate in glycolysis in the cytoplasm, where pyruvate is turned into lactate by LDHA, which then is excreted to the extracellular matrix by MCT4. Of note, lactate uptake by oxidative cells via MCT1 leads to the conversion of it back to pyruvate in the cytoplasm via LDHB, which is then transported to the mitochondria via MCT1 to complete the TAC and contributes to energy metabolism

Lactate is the end-product of glycolysis, a major substrate for oxidative metabolism, which serves as a bridge connecting many cellular pathways [15]. Lactate is transported and subsequently accumulated in different important organs via blood circulation in the body but also plays a role in regulating cellular energy and redox homeostasis by intracellular and cell-cell lactate shuttles [16]. Lactate can be exchanged between cells and the extracellular matrix and between the inner and outer mitochondrial membranes by monocarboxylate transporter (MCT) and lactate dehydrogenase (LDH) [17]. Glycolytic cells trigger a large uptake of glucose and participate in glycolysis in the cytoplasm, where pyruvate is turned into lactate by LDHA, which then is excreted to the extracellular matrix by MCT4. Of note, lactate uptake by oxidative cells via MCT1 leads to the conversion of it back to pyruvate in the cytoplasm via LDHB, which is then transported to the mitochondria via MCT1 to complete the tricarboxylic acid cycle (TAC) and contributes to energy metabolism [18]. Moreover, under stimulation with hypoxia, hydrogen peroxide, and lactate, the expression of hypoxia-inducible factor-1 (HIF-1) is upregulated in the cell, which promotes the expression of MCT4 and the exportation of lactate (Fig. 1) [19]. This lactate shuttle contributes to intercellular lactate sharing and links glycolysis with aerobic oxidation, which is conducive to more efficient allocation and exploitation of energy by tumor cells.

### Lactate: the classic and new perspectives of metabolism

Conventional wisdom suggests that glucose is the major source of nutrient supply and produces energy by two metabolic means: glycolysis and mitochondrial oxidative phosphorylation [20]. Both metabolic pathways start with pyruvate, an intermediate product from the breakdown of glucose, accompanied by the production of small amounts of ATP and NADH [21]. Under aerobic conditions, pyruvate and NADH electrons enter the mitochondria, where they are converted to acetyl-CoA, which will then go to the tricarboxylic acid (TCA) cycle to produce enormous amounts of ATP [22]. Under pathological hypoxic conditions accompanied by the failed entry of electrons into the mitochondria, pyruvate generated from glycolysis is only converted to lactic acid by LDH. Lactic acid is then dissociated into lactate and H^+^, causing the body to accumulate lactate [23]. However, this view has been currently updated and improved. Aerobic glycolysis, also known as the Warburg effect, still occurs and provides a way to quickly produce energy and lactate under stressful conditions such as tumors, exercise, trauma, sepsis, and heart failure, although cells are in an aerobic environment [6, 24]. The key mechanism of aerobic glycolysis may lie in the up-regulation of LDHA and pyruvate dehydrogenase kinase (PDK) in tumors and other states to synergistically promote the conversion of abundant pyruvate into lactate [25].

It is traditionally believed that lactate is a “metabolic waste product” and its catabolism occurs mainly in the liver, where it undergoes gluconeogenesis and reproduces glucose, a process known as the Cori cycle [26]. However, new concepts of lactate are gradually being established. Using the ^13^C-isotope tracer and metabolomics study by Jang C et al. [27], lactate has a higher circulatory turnover flux in fasted pigs despite glucose being the most abundant circulating carbohydrate, that is, the TCA cycle feeds primarily off circulating lactate, and glucose mainly provides nutrients for the TCA cycle through circulating lactate, suggesting that apparently many organs simultaneously produce and consume circulating lactate. Moreover, lactate is not only described in pigs but is also widely used as a fuel in mice and humans, confirming that lactate is a common carbohydrate fuel in mammals [28]. The ubiquitous expression of MCT and the oxidation of lactate into pyruvate for the TCA cycle in cells by LDHB also confirm that lactate has become a nearly common carbohydrate fuel [27]. Aerobic glycolysis has been intensively studied in pathophysiological processes. The increased pyruvate kinase muscle isozyme 2 (PKM2)/PKM1 ratio plays an important role in promoting the metabolic “conversion” of glucose oxidation to aerobic glycolysis, which utilizes glycolysis intermediates and upregulates the glutaminolysis, pentose phosphate pathway, and single carbon metabolism to facilitate the biosynthesis of nucleosides, thus contributing to cell proliferation [29]. The production of lactate by aerobic glycolysis has also been shown to cause a highly acidic microenvironment in the local area, which may alter immune cell infiltration to promote immunosuppression and cell proliferation [30].

### Lactate as a ligand for GPR81: a cell transduction molecule

Lactate is not only the most common carbohydrate fuel under specific physiological conditions, but also carries a deeper biological significance. It is thought that lactate acts as a ligand for the G-protein-coupled receptor 81 (GPR81), which also mediates signal transduction to facilitate the effects of lactate [31]. Lactate is involved in extracellular signal-regulated kinase (ERK) dephosphorylation by activating GPR81 and promotes cell apoptosis and susceptibility to ischemic injury in ischemic brain injury, suggesting GPR81 antagonist might be a potential strategy for brain ischemia [32]. In contrast, several studies support a possible protective role of lactate in ischemic brain damage, possibly by supplying energy to compensate for the bioenergetic crisis caused by ischemia [33, 34]. Collectively, lactate at low concentrations may exacerbate neuronal injury by activating the GPR81 receptor, while high concentrations protect nerve cells through the supply of ATP. Lactate/GPR81 pathway can also inhibit lipolysis by down-regulating cellular cAMP level, making it an important target to intervene in lipid metabolism and treat metabolic syndrome [35]. In cancer treatment, lactate/GPR81 is also required for tumour growth. On the one side, when lactate is the main energy source for tumor cells because of the Warburg effect, deletion of GPR81 results in mitochondrial functional inactivation and a marked attenuation of tumor growth [36]. On the other hand, lactate/GPR81 can promote tumor progression through multiple signaling pathways. For example, lactate-induced GPR81 activation activates the transcription factor TEAD by reducing intracellular cAMP levels, further mediating the programmed death-ligand 1 (PD-L1) promoter activation and increase of PD-L1 protein levels in lung cancer, which confirms the key role of lactate in modulating cancer cells to evade immune surveillance [37]. Another example is lactate/GPR81 signaling through activating the inosine phosphoinositide 3-kinase (PI3K)/protein kinase B (Akt)-cAMP response element binding protein (CREB) pathway, resulting in increased production of the pro-angiogenic mediator amphiregulin (AREG) to promote angiogenesis [38]. Additionally, on the plus side is the fact that increased lactate release within an infection context mediates signal transduction of the bone marrow endothelial lactate-receptor GPR81, thereby preferably promoting neutrophil mobilization by regulating the expression of endothelial VE-Cadherin and further vascular permeability in bone marrow as well as inducing the release of neutrophil mobilizers such as granulocyte colony-stimulating factor (G-CSF) [39]. Elevated lactate levels attenuate inflammation during delivery by acting on uterine GPR81 to down-regulate key pro-inflammatory genes in a feedback way, such as interleukin (IL)-1β, IL-6, chemokine ligand 2, etc. [40]. Collectively, these examples illustrate the role of the functions of lactate in ischemic damage or neuroprotection, angiogenesis, promoting tumor growth and inflammation regulation, etc.

## Protein lactylation

### Mechanisms of histone lactylation

In 2019, a mass shift of 72.021 Da in the histone lysine residues was first identified through the mass spectrometry analysis of MCF-7 cells by Zhang et al. [14], which was similar to that caused by adding a lactyl group to the ε-amino group of a lysine residue. To further corroborate this modification, they revealed lactate exposure could promote lactylation of lysine residues through metabolic labelling experiments using the isotope L-lactate (^13^C_3_) [14]. Some unique amino acid residues with substrate specificity such as lysine, arginine, and histidine present positively charged side chains at physiological pH [41]. Moreover, lysine and arginine often are located at the hydrophilic surface of proteins, and the ε-amino group of lysine and guanidinium group of arginine exposed to the solvent due to the significant hydrophobicity of these side chains are susceptible to post-translational modification [42]. Sokalingam S et al. [43] have also demonstrated that most ε-amino and guanidinium groups of lysine and arginine residues in protein structures are exposed to the solvent through the analysis of the electrostatic interactions in green fluorescent protein (GFP), which increased their potential for interactions of various physicochemical factors. Lysine is not only the most modified amino acid but also the most widely affected amino acid by PTMs in comparison with arginine, making it a hot topic in enzyme and chemical PTMs [44]. Arginine plays a major structural role in driving protein folding and stability because of its guanidinium group to form three-dimensional ionic interactions. While the geometry of lysine residues is less stable than that of arginine, its ε-amino group can form single-ion interactions, making lysine more functionally flexible and therefore easier to bring modified [45]. The relatively free coordination of ions and the chemical reactivity of the ε-amino make lysine a key component of various enzymatic catalysis. As compared to bacteria, mammals appear to have more varieties of lysine PTMs. Interestingly, lysine is one of the essential amino acids that mammals must obtain from the diet, which makes lysine PTMs nutrient-sensitive and sense cell metabolic states to varying degrees [45]. Thus, the nature of lysine makes these related PTMs play a crucial role in the regulation of cell health.

**Fig. 2:**
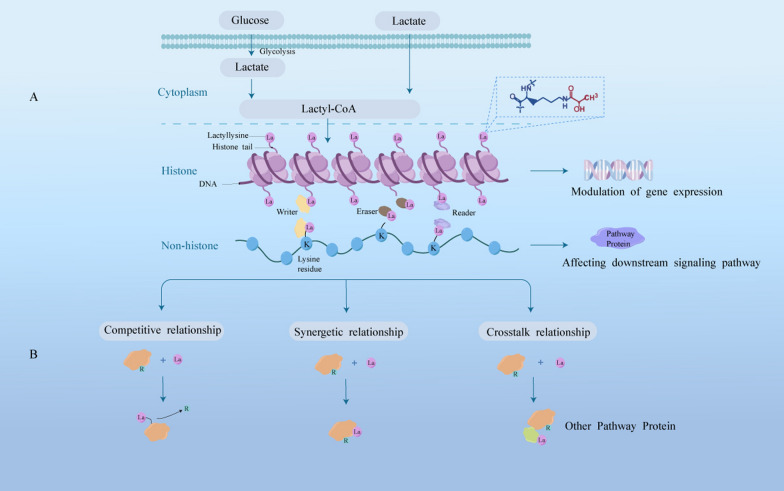
Lactate from extracellular matrix or glycolysis resulted in lactylation. **A** Lactate may synthesize lactyl-CoA, and then, the lactyl group is transferred by “writer” to lysine, leading to lactylation of histones or non-histones to affect gene expression or downstream signaling pathways. **B** Three types of interactions (in different color blocks) between lactylation (pink residues with La) and other PTMs (green residues with R) are shown

Exogenous and endogenous L-lactate, but not D-lactate, accumulates to a certain extent and directly promotes lactylation of specific lysine residues [46]. Glycolysis inhibitors directly correlate with both decreased lactate production and lysine lactylation (Kla), while mitochondrial inhibitors and cellular hypoxia may increase lactate production and enhance Kla [47]. In the majority of investigated lactylation modified proteins, lactyl moieties of lactyl-CoA from L-lactate are bound via the ε-amino group of lysines to the target protein. Generally, this process starts with relevant enzymes. First, the “writers”, a series of specific acylases, transfers the lactyl group of lactyl-CoA as a substrate to a histone or non-histone lysine residues, which alters the protein structure and function. Then, “erasers” emerge to stop the entire Kla cycle and prevent the protein lysine from having lasting effects, meaning that they act as deacylases to remove part or all of the lactyl groups from the target proteins. Finally, effector proteins called “readers” specifically recognize this change in Kla to affect downstream signaling pathways and initiate various biological events (Fig. 2) [48]. Moreover, non-enzymatic lysine acylation involves the deprotonation of the ε-amino group of the substrate lysine by a general base, such as aspartate or glutamate. Then, the deprotonated ε-amino group initiates the nucleophilic attack on the thioester bond of acyl-CoA greatly [49]. Importantly, the terminal carboxylic acids of acyl-CoA form a highly reactive cyclic anhydride intermediate through intramolecular catalysis and react strongly with free lysine ε-amino to produce non-enzymatic acyl-lysine modifications [50]. However, lactyl-glutathione (LGSH), but not lactyl-CoA, as in conventional studies, has been reported to be involved in another unique non-enzymatic lactyl transfer [51]. A typical non-enzymatic acyl-transfer mechanism occurs in acetyl-glutathione, whose acetyl group is transferred to the ε-amino group of a lysine residue to generate lysine acetylation (Kac) [52]. Gaffney DO et al. [51] further discussed the non-enzymatic kla based on LGSH considering the chemical similarity between acetyl-glutathione and LGSH. The main conclusions are listed below: methylglyoxal, a reactive glycolytic by-product, rapidly binds to glutathione via glyoxalase 1 (GLO1) to produce LGSH, which transfers its own lactyl group to a protein lysine residue in a non-enzymatic acyl-transfer mechanism. And, LGSH is also hydrolyzed by glyoxalase 2 (GLO2) to recycle glutathione and produce D-lactate. Thus, GLO1 amplification without an accompanying compensatory increase in GLO2 may predispose the balance of the glyoxalase cycle towards LGSH and further kla production. Interestingly, glycolytic proteins are major targets for Kla through the feedback regulation, presented with inhibition of glycolytic enzyme activity and reduction of glycolytic metabolites [51]. Overall, knowledge regarding Kla as a novel PTM remains limited, particularly for the substrates, modification reactions (enzymatic or non-enzymatic), and lactylation dynamics.

### Crosstalk between lactylation and other acylations

There is potential crosstalk among lysine acylations because they are intertwined in the metabolic networks of cells. Profoundly understanding the crosstalk in PTMs may be helpful for further mining of lactylation modification. Therefore, attention must be paid to the metabolic pathways that are interrelated and regulated when explaining the relationship between lactylation and other acylations [53]. Most proteins function through interactions with other proteins. It is reported that many proteins have at least one PTM, and many of them have more than one, suggesting crosstalk among different PTMs of proteins is ubiquitous [54]. In particular, there is a high degree of similarity and coordination between lactylation and acetylation, which are important processes linking metabolism and epigenetics [55]. For instance, two types of PTMs tend to target lysine and hold some enzymes in common, e.g., p300 as the writer [14]. Li L et al. [56] showed that Gli-like transcription factor 1 (Glis1) enhanced levels of acetyl-coA and lactate as well as synergistically drove histone acetylation and lactylation by transcriptional activation of glycolytic genes and higher glycolytic flux. Moreover, lactate may act as an important transcriptional regulator and induce histone hyperacetylation by promoting expression of histone deacetylase (HDAC)-associated genes to inhibit HDAC activity [57]. Interestingly, several studies have shown that a portion of histone acetyltransferases (HATs) and HDACs catalyze the lactylation and delactylation of histones, respectively [14, 46]. Since lactation and acetylation are subject to the regulation of both HATs and HDACs simultaneously, it is reasonable to ascertain the correlation between them [46, 58]. The link between lactylation and acetylation has been shown in more studies. For instance, lactate simultaneously promotes the lactylation and acetylation of high mobility group protein B1 (HMGB1) in macrophages by activating p300 acetylase and inhibiting the activity of SIRT1 deacetylase [59]. Additionally, cold exposure can trigger metabolic reprogramming of aerobic glycolysis driven by mitochondrial damage in macrophages, increase histone acetylation to promote the release of inflammatory factors. In turn, the accumulation of intracellular lactate results in histone lactylation as a self-protective mechanism to initiate the transcription of anti-inflammatory genes [60]. However, the changes of Kla and Kac differ across cell types. Under hypoxia condition, both human Hela cells and murine macrophages show increased Kla levels, but Kac levels decrease in the former, and remain unaffected in the latter [14]. It is for this reason that the changes of Kla and Kac in different cells responded to different stimulations are not always consistent. Therefore, simply attributing the relationship between Kla and Kac or other acylation modifications to synergy or competition seems unreasonable.

In addition to acetylation, other posttranslational acylations, such as succinylation, crotonylation and butyrylation, have also been reported to crosstalk with lactylation. For instance, succinylation of PKM2 at lysine residue K311 in LPS-induced macrophages helps PKM2 enter the nucleus to promote the expression of IL-1β and HIF-1α-dependent genes and the metabolic shift to aerobic glycolysis (lactate production) [61, 62]. In contrast, SIRT5 acts as an “eraser” of succinylation, effectively desuccinylates and activates PKM2, thereby reversing the above process [61]. Moreover, histone lysine crotonylation (Kcr) and Kla are distributed widely throughout the brain, and HDACs have been shown to “erase” histone Kcr and Kla. While inhibition of HDACs stimulates the levels of histone acylation modifications (H3K9cr and H3K18la) in vivo and in vitro, and widely promotes neuronal differentiation and cell proliferation processes [63]. Lactylation could also be associated with butyrylation mediated by butyrate, which contributes to increased levels of protein Kla in human Hela cells, and may be prevented by inhibition of HDACs [64]. Overall, lactylation may be related to other acylation modifications in ways we do not yet understand, including other propionylation, glutarylation, betahydroxybutyrylation, and 2-hydroxyisobutyrylation [65–68].

## Lactylation in health and diseases

Protein lactylation has been extensively detected and studied in various disease models. Lactate accumulation from metabolic reorganization in multiple diseases controls the progress of disease. At the same time, lactate and histone lactylation also seem to be highly necessary for neurodevelopment and to orchestrate gene expression changes [63]. The role of histone or non-histone lactylation in neuronal development, cancer, inflammation, embryogenesis, cerebral disease, fibrosis, and so on will be discussed in the following sections (Table 1).

**Table 1:** The function of lysine lactylation modification in physiology and disease

Study/Years of publication	Protein and Kla sites (if available)	Cell lines and animal models (if available)	Mechanisms and effects	Intervention and dose
Dai SK et al., 2022 [63]	Histone H3 (K18)	P19 EC, neural stem/progenitor and 293T cells; ICR/CD−1 mice	Histone Kla existed extensively and changed significantly in the developing telencephalon to orchestrate gene expression changes and widely participate in neuronal differentiation and cell proliferation processes. HDAC1−3 acted as a novel “eraser” of H3K18la, affecting H3K18la levels	SAHA: 0.5 µM (for cells); VPA: 1 mM (for cells); MS−275: 1 µM (for cells), 20 mg/kg (for models); EX 527: 1 µM (for cells)
Nian F et al., 2022 [73]	Histone H3 (K18)	HEK293FT and MC3T3-E1 cells	The increased expression levels of LDHA and its related H3K18lac during osteoblast differentiation promoted the formation of cellular mineralized nodules and ALP activity and played an important role in osteoblast differentiation	LA (unmentioned dosage)
Wang J et al., 2022 [76]	Histone H4 (K8)	HCT116, HEK293T, RAW 264.7 and FHC cells infected with *S. typhimurium*	LPS decreased the negative regulatory efficiency of YY1 on LINC00152 through histone lactylation, thereby up-regulating LINC00152, which inhibited both Salmonella invasion and the inflammation response but also promoted tumor growth	Lactic acid: 25, 50, 75, 100 mM (for cells); LPS: 1, 5, 10 µg/ml
Irizarry-Caro RA et al. 2020 [75]	Histone	Primary BMDMs isolated from femur and tibia; BCAP^ΔMϕ^ mice that specifically lack BCAP in macrophages	Macrophage-specific deletion of BCAP led to reduced histone lactylation through deficiency in aerobic glycolysis and lactate production and therefore attenuated expression of reparative macrophage genes such as ARG1 and KLF4	LA: 25 mM (for cells)
Zhang D et al., 2019 [14]	Histone H3 (K18)	MCF−7, MDA-MB−231, HeLa, A549, HepG2, MEF, and RAW 264.7 cells; Ldha^fl/fl^ mice with LysMcre^+/−^ or LysMcre^−/−^	Hypoxia and bacteria induced production of lactate through glycolysis, which stimulated histone lactylation and further promoted M2-like characteristics and the expression of homeostatic genes involved in the damage repair process	Glucose: 0, 1, 5, 25 mM (for cells); DCA: 0, 5, 10, 20 mM (for cells); Oxamate: 0, 5, 10, 20 mM (for cells); Rotenone: 0, 5, 10, 50 nM (for cells)
Yao X et al., 2023 [85]	Histone H3 (K18)	N2a cells stimulated by OGD/R; Male Sprague Dawley rats as cerebral infarction models via the MCAO method	Upregulated LDHA facilitated the expression of IL−18 and IL−1β, and also induced cell pyroptosis by promoting the lactylation of histone H3K18la to upregulate the level of HMGB1. Conversely, LDHA knockdown relieved the cerebral infarction injury in the MCAO rat model	LA: 15 µM (for cells)
Hagihara H et al., 2021 [87]	Histone H1	Primary neuronal cells separated from C57BL/6J mice brains; C57BL/6J mice and ICR mice	Stress-associated neural excitation and social defeat stress increased brain lactate and histone lactylation levels, which was associated with a decrease in social behavior and an increase in anxiety-like behavior	LA: 2, 5, and 25 mM (for cells); Oxamate: 1 g/kg (for models); 4-CIN: 0.01 and 1 mM (for cells)
Pan RY et al., 2022 [86]	Histone H4 (K12)	Primary microglia separated from mouse brains, and microglial BV2 cell line; Microglia-specific Pkm2 knockout mice as an Alzheimer’s disease model induced by tamoxifen	Elevated lactate and histone lactylation levels in AD further promoted the expression of glycolytic gene PKM2, thus forming a positive feedback loop that contributes to the abnormal activation and dysfunction of microglia	LA: 20 mM (for cells); Shikonin: 0.5 µM (for cells) and 0.5 mM (for models); compound 3 K: 5 µM (for cells) and 1 mM (for models)
Li X et al., 2022 [88]	Histone H3 (K18)	HTR−8/SVneo and TEV−1 cells exposed to hypoxia	Hypoxia increased the expression of fibrosis-related genes, FN1 and SERPINE1, through lactate-induced histone lactylation in trophoblast cells	LA: 0, 1, 5, 25 mM (for cells); Lactic acid: 0, 1, 5 mM (for cells); Oxamate: 0, 5, 10, 20 mM (for cells)
Cui H et al., 2021 [89]	Histone	Primary mouse alveolar macrophages and BMDMs; Male C57BL/6 mice as lung fibrosis models via intratracheal instillation of bleomycin or adenovirus expressing TGF-β1	TGF-β1 stimulated the increase of lactate production in myofibroblasts and secreted it into the extracellular milieu to promote histone lactylation in macrophages, thereby inducing the expression of some profibrotic mediators. And, p300 as a “writer” mediated lactate-induced histone lactylation	LA: 20 mM (for cells)
Wang N et al., 2022 [91]	Histone H3 (K18)	Mouse aortic endothelial cells, and primary mouse peripheral blood monocytes and cardiac fibroblasts; Male C57BL/6 mice as MI models via ligation of the left coronary artery	Histone lactylation in monocytes increases rapidly after MI and promoted activation of reparative genes Lrg1, Vegf-a, and IL−10, which is conducive to the reparative environment and the improvement of cardiac function after MI. GCN5, as a writer of histone lactylation, promoted H3K18la in an IL−1β-dependent manner	LA: 20 mM (for cells); Rotenone: 5 nM (for cells); FX−11: 40 µM (for cells); DCA: 5 mM (for cells)
He Y et al., 2023 [99]	Histone H3 (K18)	LNCaP, NCI-H660, LAPC−4, VCaP, A549, LNCaP/AR, PC9/ER and NCI-H1688 cells; Pbsn-Cre4 mice models	NEPC exhibited preferential utilization of aerobic glycolysis due to an impairment of the Parkin-mediated mitophagy, which subsequently led to histone lactylation and upregulation of transcription of neuroendocrine associated genes. However, the cell fate determinant Numb reversed this process by binding to Parkin	DCA or oxamate: 10 mM (for cells); CCCP: 10 mM (for cells); Rapamycin: 10 nM and 50 nM (for cells)
Jiang J et al., 2021 [100]	Histone H3	Human lung bronchial epithelial cell line BEAS−2B, NSCLC cell lines including A549 and H1299	Lactate regulated cellular metabolism at least in part through up-regulation of HK−1 and IDH3G gene expression mediated by histone lactylation in NSCLC cells	LA: 5 or 10 mM (for cells)
Yang J et al., 2022 [101]	Histone H3 (K18)	Human renal epithelial cell HK2, and human RCC cell lines 786-O, A498, Caki−1 and ACHN; BALB/c nude mice and NSG mice via subcutaneous injection of sectioned ccRCC tissues	Inactive VHL induced histone lactylation in a HIFs-dependent manner, thereby transcriptionally activating the expression of PDGFRβ to promote ccRCC progression. In addition, overexpression of PDGFRβ positively stimulated histone lactylation	Oxamate: 8 mM (for cells), 60 mg/kg (for models); 2-DG: 4 mM (for cells)
Pan L et al., 2021 [102]	Histone H3 (K9/56)	LCSCs, human hepatoma cell lines including HCCLM3 and Hep3B; Female nude mice via subcutaneous injection of 3B-LCSCs cells	DML reduced the lactate level of LCSCs by regulation of the glycolytic metabolic pathway to attenuate histone lactylation, thus playing an anti-cancer role	NA
Yu J et al., 2021 [103]	Histone H3 (K18)	PIG1 and HEK293T cells, and human melanoma cell lines (92.1, MUM2B, OCM1, MEL290, OMM1, CRMM1, CRMM2, and CM2005.1); Male BALB/c nude mice via choroid injection of ocular melanoma cells	Elevated histone lactylation (modulated by EP300, a “writer”) effectively promoted the tumorigenesis of ocular melanoma through up-regulating the transcription of YTHDF2 and further inducing the degradation of PER1 and TP53 mRNAs via binding to their respective m6A sites	LA: 1, 5, and 25 mM (for cells); Oxamate: 5, 10, and 20 mM (for cells); 2-DG: 1, 10, and 20 mM (for cells)
Xiong J et al., 2022 [104]	Histone H3 (K18)	HEK293T, LLC, B16F10, and MC38 cells, and primary bone marrow cells and MDSCs; C57BL/6 mice via subcutaneous injection of MC38, B16F10, or LLC cells	Elevated lactate in tumor-infiltrating myeloid cells induced METTL3 expression by promoting histone lactylation, and further m6A modification on Jak1 mRNA, which promoted its protein translation and strengthened downstream STAT3 signal that enhanced immunosuppressive functions of myeloid cells	LA: 25 mM (for cells); C646: 100 µM (for cells); Trichostatin A: 1 µM (for cells)
Yang Q et al., 2022 [106]	Histone H3 (K18)	Ishikawa cells; Chinese Small Tail Han ewes as pregnancy models	During pregnancy, increased levels of H3K18 lactylation and lactate helped to maintain glutathione-based redox homeostasis and apoptotic balance, which are essential for successful embryo implantation	LA: 0.04, 0.2, 1, 5, 10, 50, 100 mM (for cells); Oxamate: 10, 25 mg/ml (for cells)
Chen J et al., 2023 [109]	Histone H3 (K18)	Primary PASMCs isolated from from adult male Sprague Dawley rats; Male Sprague Dawley rats as pulmonary hypertension models via exposure to hypoxia	Hypoxia-induced mROS further triggered lactate accumulation and histone lactylation in PASMCs by upregulating HIF−1α/PDK1&2/p-PDH-E1α axis, which promoted the proliferation of PASMCs	LA: 10 mM (for cells); DCA: 10 mM (for cells); Oxamate: 50 µM (for cells), 750 mg/kg (for models)
Yang W et al., 2021 [107]	Histone H3 (K18/23)	Oocytes/embryos collected from ICR mice	Inhibition of LDHA activity reduced histone lactylation, thereby impairing embryonic pre-implantation development	GSK2837808A: 100 pmol (for cells)
Wang J et al., 2022 [77]	PKM2 (K62)	Primary mouse BMDMs separated from C57BL/6 mice femurs; Male C57BL/6 mice made with two full-thickness skin wounds (9-mm) on the back	Lactate increased the lactylation level of PKM2 and inhibited its tetramer-to-dimer transition, activating pyruvate kinase and promoting the transition of LPS-induced macrophages to a reparative phenotype	LA: 20 mM (for cells and models)
Yang K et al., 2022 [59]	HMGB1	RAW 264.7 macrophages; Macrophage-specific YAP knockout mice as a polymicrobial sepsis model via cecal ligation and puncture	Elevated lactate levels during polymicrobial sepsis promoted the secretion of HMGB1 from macrophage exosomes and its lactylation in a p300/CBP-dependent mechanism, further inducing endothelial dysfunction	LA: 10 mM (for cells); Oxamate: 20 mM (for cells)
Zhang W et al., 2023 [83]	LCP1	Neurocytes (PC12) stimulated by OGD/R; Male Sprague Dawley rats as cerebral infarction models via the MCAO method	The elevated lactylation of LCP1 in cerebral infarction reduced its own degradation and cell viability and enhanced the apoptosis rate of PC12 cells. However, LCP1 knockdown or inhibiting the glycolysis relieved the cerebral infarction injury	LA: 15 µM (for cells); 2-DG: 20 mM (for cells)
Fan M et al., 2023 [90]	Snail1	HUVEC cells and HCMECs; Sex-matched C57BL/6 mice as MI models via MI surgery	After MI, high lactate levels induced the association between p300/CBP and Snail1, which promoted the lactylation of Snail1 and thereby activated the TGF-β/Smad2 pathway and further endothelial-to-mesenchymal transition	2-DG: 0.5 g/kg (for models); LA: 5 or 10 mM (for cells), 0.5 g/kg (for models)
Yao G et al., 2023 [94]	c-myc	HCC cell lines including Huh7 and HCCLM3 exposed to hypoxia	GPC3 knockdown alleviates viability, migration, invasion and glycolysis of HCC cells by inhibiting the lactylation of c-myc to promote itself degradation	LA: 15 µM (for cells); 2-DG: 10 mM (for cells)
Miao Z et al., 2023 [96]	β-catenin	CRC cell lines including SW620 and RKO exposed to hypoxia; Male BALB/c nude mice via subcutaneous injection of infected SW620 cells	Hypoxia-induced glycolysis promotes the lactylation of β-catenin in CRC cells to further enhance the protein stability and expression of β-catenin, thereby aggravating the progression of CRC through the Wnt signaling pathway	LA: 15 µM (for cells); 2-DG: 10 mM (for cells); Wnt agonist1: 20 µM (for cells)
Luo Y et al., 2022 [97]	HIF−1α	PC−3 and DU145 cell lines; Male BALB/c nude mice via subcutaneous injection of infected PC−3 cells	Elevated lactate in prostate cancer promoted lactylation of HIF1α to induce KIAA1199-mediated angiogenesis, vasculogenic mimicry and depolymerized hyaluronic acid levels	LA: 10 mM (for cells)
Yang Z et al., 2023 [95]	AK2 (K28)	HepG2 or Hep3B cells	High lactylation of AK2 in HCC could significantly reduce its own activity, mediate perturbation of ATP metabolism and down-regulate the intrinsic apoptosis pathway to promote cancer cell proliferation and migration, and predict poor prognosis in HCC patients	LA: 10 mM (for cells); Glucose: 10, 25 mM (for cells)
Gu J et al., 2022 [98]	MOESIN (K72)	Naive CD4 ^+^ T and CD3 ^+^ T cells separated from murine leukocytes from lymph nodes and spleens; Male C57BL/6 mice via axillary injection of mouse Hepa1−6 tumor cells	Lactylation of MOESIN at Lys72 enhanced TGF-β and downstream SMAD3 signaling in Treg cells through TGF-βRI to regulate the development and function of Treg cells to control tumorigenesis and antitumor therapy	LA: 10, 20 mM (for cells); GSK2837808A: 20 mg/kg (for models)
Sun Y et al., 2022 [105]	PARP1 (K498/505/506/508/518/521/524)	HEK293T cells	Hyperlactylation of PARP1 regulated its ADP-ribosylation activity and may contribute to DNA repair	LA: 10 or 25 mM (for cells)
Wang X et al., 2023 [110]	YY1 (K183)	HMC3 cells exposed to hypoxia; Male and female C57BL/6 J mice as OIR models via placing them into a glass oxygen chamber	Hyperlactylation of non-histone YY1 under hypoxia was regulated by p300 as a “writer”. YY1 was directly bound to the promoter of FGF2 and promoted the transcription of FGF2 through its high lactylation, thus promoting the formation of neovascularization. This situation was reversed by the p300 inhibitor A−485	DCA: 1.5 mg/kg/day (for models), 20 mM (for cells); Rotenone: 200 mg/kg/day (for models), 50 nM (for cells); A−485: 5, 10, and 20 µM (for cells)
Gao R et al., 2023 [108]	FASN (K673)	AML−12, HepG2, 293T and RAW 264.7 cells; Male MPC1+/- and wildtype C57/BL6J mice fed with a HFD or a LFD	MPC1 knockout induced lactate accumulation, promoted the lactylation of FASN in hepatocytes, and lactylation at the K673 site of FASN inhibited own activity, thereby mediating the down-regulation of liver lipid accumulation	GSK2837808A: 10 µM (for cells)

*ALP* alkaline phosphatase,* AK2* adenylate kinase 2,* ARG1* arginase−1,* CBP* CREB-binding protein,* DML* demethylzeylasteral,* DCA* dichloroacetate,* BMDMs* bone marrow-derived macrophages,* BCAP* B-cell adapter for PI3K,* ccRCC* clear cell renal cell carcinoma,* FASN* fatty acid synthase,* GCN5* general control non-depressible 5,* GPC3* glypican−3,* HIF−1α* hypoxia-inducible factor−1α,* HIFs* hypoxia-inducible factors,* HCMECs* human cardiac microvascular endothelial cells,* HMGB1* high mobility group box−1,* HFD* high-fat diet,* HCC* hepatocellular carcinoma, * KLF4* Krüppel like factor−4,* K* lysine,* Kla* lysine lactylation,* LPS* lipopolysaccharide,* LCSCs* liver cancer stem cells,* LDHA* lactate dehydrogenase A,* LA* lactate,* LCP1* lymphocyte cytosolic protein 1,* LFD* low-fat diet,* MCAO* middle cerebral artery occlusion,* MPC1* mitochondrial pyruvate carrier 1,* MDSCs* myeloid-derived suppressor cells,* MI* myocardial infarction,* mROS* mitochondrial reactive oxygen species,* NEPC* neuroendocrine prostate cancer,* NA* not available,* NSCLC* non-small cell lung cancer,* OGD/R* oxygen-glucose deprivation/reoxygenation,* OIR* oxygen-induced retinopathy,* PARP1* poly ADP-ribose polymerase 1,* PKM2* pyruvate kinase M2,* PASMCs* pulmonary artery smooth muscle cells,* PDGFRβ* platelet-derived growth factor receptor β,* PDK1&2* PDH kinase 1 and 2,* p-PDH-E1α* phosphorylation of PDH-E1α,* TGF-β1* transforming growth factor-β1,* TGF-βRI* transforming growth factor β receptor I,* VHL* von Hippel-Lindau,* 4-CIN* α-cyano−4-hydroxycinnamate

**Table 2:** Therapeutic approaches targeting the lactate/lactylation axis

Target	Representative drugs	Research status	Example diseases	Effects	Ref/Trial No.
MCT1	AZD3965	Clinical trial	Raji B cell lymphoma	Lactate uptake	NCT01791595
MCT1	BAY8002	Pre-clinical	Triple negative breast cancer	Lactate uptake	[118]
MCT1	SR13800	Pre-clinical	Neuroblastoma, lyphoma, breast cancer	Lactate uptake	[119]
MCT1	AR-C155858	Pre-clinical	Breast cancer, colorectal cancer	Lactate uptake	[116]
MCT1	7ACC2	Pre-clinical	Pancreatic cancer	Lactate uptake	[117]
MCT4	Syrosingopine	Pre-clinical	Myeloma	Lactate excretion	[115]
LDHA	GSK2837808A	Pre-clinical	Liver cancer	Lactate production	[98]
LDHA	Oxamate	Pre-clinical	Prostate and lung adenocarcinomas	Lactate production	[99]
PDHK	Dichloroacetate	Clinical trial	Metastatic breast cancer, lung cancer, etc.	Lactate production	NCT01029925, NCT01386632, etc.
Hexokinase	2DG	Clinical trial	Prostate cancer, lung cancer, breast cancer, etc.	Lactate production	NCT00096707, NCT00588185, etc.
Hexokinase	3-BrPA	Pre-clinical	Breast cancer, liver cancer, bladder cancer, etc.	Lactate production	[122]
Hexokinase	Tristetraprolin	Pre-clinical	Breast cancer	Lactate production	[123]
Hexokinase	Lonidamine	Pre-clinical	Lung cancer, breast cancer, melanoma, etc.	Lactate production	[124]
GPR81	Curcumin	Clinical trial	Acute lymphoblastic leukemia, prostate cancer, etc.	Lactate uptake	NCT05045443, NCT04731844, etc.
GPR81	LRH7-G5	Pre-clinical	Triple negative breast cancer	Lactate uptake	[118]
HDAC	ITSA-1	Pre-clinical	Nasopharynx cancer	Lactylation production	[126]
HAT	Garcinol	Pre-clinical	Breast cancer, colon cancer, lung cancer, etc.	Lactylation production	[127]
GCN5	CPTH6	Pre-clinical	Leukemia, lung cancer	Lactylation production	[128]
p300/CBP	A-485	Pre-clinical	Neovascularization, pituitary adenoma, melanoma, etc.	Lactylation production	[110]

*GPR1* G-protein-coupled receptor 81,* GCN5* general control non-derepressible 5,* HAT* histone acetyltransferase,* HDAC* histone deacetylase,* LDHA* lactate dehydrogenase A,* MCT* monocarboxylate transporter,* PDHK* pyruvate dehydrogenase kinase,* 2DG* 2-deoxy-D-glucose,* 3-BrPA* 3-Bromopyruvic acid

### Cellular development and differentiation

Histone lactylation that marks numerous genes is widely distributed throughout the developing telencephalon and changes dynamically in the course of development, indicating histone lactylation is an intrinsic pathway to regulate gene expression during mammalian development [63]. There is a metabolic transition from glycolysis to mitochondrial oxidative phosphorylation during neurogenesis, which contributes to lower levels of lactate and lactyl-CoA and could have affected whole histone Kla levels during development [69]. Dai SK et al. [63] showed that the levels of H3K18la and even total histone H3 Kla declined over time during neurogenesis and differentiation in mice, while the increased levels of multiple histone lactylations pre-activated neuronal transcriptional programs and promoted the differentiated maturity of neural stem cells by the inhibition of “eraser” HDAC1-3. In contrast, p300/CBP acts as a “writer” of histone lactylation, and its knockdown inhibits embryonic neural differentiation in the normal and Rubinstein-Taybi syndrome brain [70]. Similarly, genes associated with neural development and differentiation remain primed in the early stages of neurogenesis, and HATs and HDACs separately promote the lactylation and delactylation of histones that target these primed genes and thus regulate neurogenesis [71, 72]. In fact, the switch for histone lactylation depends on the balance between “writers” (such as CBP/p300 and HATs) and “erasers” (such as HDACs) and acts as regulatory elements of genes determining neural fate. Overall, the crosstalk of multiple histone acylations, more than just lactylation, plays a key role in the regulation of neural development and disease. Another is that glucose tends to be metabolized most to produce lactate through aerobic glycolysis during osteogenic differentiation, characterized by elevated LDHA levels [73]. JunB, a component of the activator protein-1 (AP-1) transcription factor family, is involved in osteoblast differentiation and bone formation [74]. The lactate-derived histone H3K18la levels gradually increase and are remarkably enriched on the promoter of JunB to activate its expression, which contributes to the formation of mineralized nodules and alkaline phosphatase activity [73]. Moreover, lactate supplementation also facilitates transcriptional elongation through enhanced histone lactylation on germline and embryo cleavage-related genes, which induces global up-regulation of genes involved in embryo cleavage [55] (Table 2)

### **Inflammation**

There is growing evidence suggesting that histone or non-histone lactylation is strongly associated with inflammation [14, 59, 75–77]. Current research on lactylation associated with inflammation mainly focuses on macrophages, which are highly plastic cells of the innate immune system and could promote or resolve inflammation under different functional phenotypes [78]. In the colitis model, the toll-like receptor (TLR) stimulated by LPS activates PI3K-Akt in a B-cell adapter for PI3K (BCAP)-dependent manner, which further leads to the accumulation of lactate and histone lactylation and therefore enhances expression of reparative macrophage genes associated with the M2-like phenotype, such as ARG1 and KLF4 [75]. Conversely, the loss of BCAP may exaggerate the inflammatory response following TLR activation. Another, lactate inhibits its tetramer-to-dimer transition and nuclear distribution as well as thus activates PKM2 by promoting the lactylation level of PKM2 at the K62 site, ultimately inducing a macrophage phenotypic switch toward reparative M2 macrophages, manifested by decreased expression of inflammatory factors [77]. Similarly, macrophages could also enhance the uptake of extracellular lactate via MCT during polymicrobial sepsis with elevated lactate levels and promote the lactylation of HMGB1 dependent on the “writer” p300/CBP. And, then, HMGB1 with elevated lactylation levels in macrophages is more released and accumulated into the cytoplasm via exosome secretion to further induce endothelial barrier dysfunction [59]. Concerning histone lactylation, which is most widely studied, hypoxia and bacterial challenges boost lactate production and elevated histone H3 lactylation at the K18 site by glycolysis in the late phase of M1 macrophage polarization, which induces the expression of genes involved in the damage repair homeostasis [14]. Significantly, histone lactylation and acetylation have different temporal kinetics, and histone lactylation occurs later than acetylation, which explains the expression of repair genes in the late phase of M1 macrophage polarization to promote homeostasis. Moreover, multiple studies have shown that long non-coding RNAs (lncRNAs) play a crucial role (such as host immune response and pathogen transmission) in pathogenic infections [79]. LPS treatment and bacterial infection upregulate the expression of LINC00152 in human colon cell lines by introducing histone lactylation on its promoter, and decreasing the binding efficiency of repressor YY1 to it, which resists both Salmonella invasion and the inflammatory response [76].

### Brain diseases

Lactate has been proposed as an energy substrate source for neurons and a valuable cell-cell signaling molecule in the brain, which is linked to neurological and psychiatric diseases [80]. The abnormal expression of lymphocyte cytosolic protein 1 (LCP1) is closely related to various cancer stages and severity [81]. Recently, Wen et al. [82] showed that LCP1 was significantly up-regulated on the 14th day in the middle cerebral artery occlusion (MCAO) rat model through proteomic analysis. The elevated lactylation levels of LCP1 by excessive glycolysis in cerebral infarction reduce its own degradation and cell viability, and enhance the cell apoptosis rate in vitro, and increase the brain water content, infarct area, and neurological score in vivo [83]. However, LCP1 knockdown or inhibiting the glycolysis reverses the above process and relieves the cerebral infarction injury [83]. HMGB1 is typically loosely bound to DNA in the nucleus but released into the cytoplasm or extracellular space when cells are damaged by external stimuli, inducing apoptosis and inflammatory responses [84]. Yao X et al. [85] showed that upregulated LDHA increased the lactate content and promoted the lactylation of histone H3K18la, which was significantly enriched on the HMGB1 promoter and upregulated HMGB1 expression, hence inducing cell pyroptosis and aggravating cerebral ischemia-reperfusion injury. Similarly, elevated lactate and histone H4K12la levels also are observed in Alzheimer’s disease and further promote the expression of glycolytic gene PKM2, thus forming a positive feedback loop that contributes to the abnormal activation and dysfunction of microglia as well as neuroinflammation [86]. Interruption of this loop by blocking PKM2 could ameliorate microglial dysfunction and Aβ pathology [86]. Interestingly, stress-associated neural excitation and social defeat stress also increase lactate and histone H1 lactylation levels in the brain, which is associated with a decrease in social behavior and an increase in anxiety-like behavior [87].

### Fibrosis

There is compelling evidence that the upregulation of glycolysis in trophoblast cells, macrophages, and myocardial endothelial cells contributes to the progression of placental, pulmonary, and myocardial fibrosis, respectively [88–90]. Reduced blood flow to the uteroplacental unit in preeclampsia leads to a hypoxic condition in the placenta, which in turn promotes excessive lactate production by trophoblast cells and induces histone lactylation to regulate the expression of genes associated with preeclamptic placental fibrosis (FN1 and SERPINE1) [88]. Another, TGF-β1 (transforming growth factor-β1) stimulates the increase of lactate production in lung myofibroblasts and secrete it into the extracellular milieu to promote histone lactylation in the promoters of the profibrotic genes in macrophages, thereby inducing the expression of some profibrotic mediators [89]. Additionally, after myocardial infarction, high lactate levels induce lactylation of Snail1, a TGF-β transcription factor, thereby activating the TGF-β/Smad2 pathway to further up-regulate endothelial-to-mesenchymal transition and exacerbate cardiac dysfunction and fibrosis [90]. And, p300 as a “writer” mediates lactate-induced lactylation of histone and Snail1 in these processes. Intriguingly, Wang N et al. [91] showed that GCN5, as a writer of histone lactylation, promoted Histone H3 lactylation in monocytes in an IL-1β-dependent manner after myocardial infarction and activated reparative genes Lrg1, Vegf-a, and IL-10, which is conducive to the reparative environment and the improvement of cardiac function. Overall, these findings shed light on the mechanism underlying the key contribution of lactate and lactylation to the pathogenesis of different fibrotic diseases.

### Tumors

The tumor microenvironment is often characterized by lactate, a core metabolite produced by the Warburg effect [92]. In the last decades, lactate may be considered a biological marker of malignancy, and it was found to be strongly associated with shorter overall survival and a higher incidence of metastasis in tumor patients [93]. This association led us to question whether lactate has a role in cancer progression. The available data already suggest tumor-associated lysine lactylation occurs on both histone and non-histone proteins. In hepatocellular carcinoma (HCC), glypican-3 knockdown reduces the lactylation of c-myc and further reduces the protein stability and expression of c-myc, thereby inhibiting the progression of liver cancer [94]. Another example is that high lactylation of adenylate kinase 2 in HCC could significantly reduce its own activity, mediate perturbation of ATP metabolism and down-regulate the intrinsic apoptosis pathway to promote cancer cell proliferation and migration, and predict poor prognosis in HCC patients [95]. Concerning colorectal cancer (CRC), Hypoxia-induced glycolysis promotes the lactylation of β-catenin to further enhance the protein stability and expression of β-catenin, ultimately aggravating the progression of CRC through the Wnt signaling pathway [96]. In prostate cancer, elevated lactate promotes the lactylation and stability of HIF1α to induce KIAA1199 transcription and KIAA1199-mediated angiogenesis, vasculogenic mimicry and depolymerized hyaluronic acid levels [97]. Moreover, Gu J et al. [98] showed that the lactylation of MOESIN at lys72 enhanced TGF-β and downstream SMAD3 signaling in Treg cells through TGF-β receptor I to regulate the development and function of Treg cells to increase tumorigenesis and tumor growth.

In addition, histone H3 (e.g., K9, K18, and K56) are also found to be involved in the regulation of various cancer types including lung, prostate, kidney, colon, liver, and melanoma [99–104]. He Y et al. [99] demonstrated that prostate and lung adenocarcinomas exhibited preferential utilization of aerobic glycolysis and concomitant histone hyperlactylation due to an impairment of the Parkin-mediated mitophagy, which subsequently led to the metabolic reprogramming and neuroendocrine differentiation following upregulation of neuroendocrine gene expression. However, the cell fate determinant Numb reversed this process by binding to Parkin. Lactate also regulates cellular metabolism at least in part through down-regulating HK-1 (glycolytic enzyme) and up-regulating IDH3G (TCA cycle enzyme) gene expression mediated by histone lactylation in non-small cell lung cancer [100]. In clear cell renal cell carcinoma, inactive von Hippel-Lindau (VHL) induces histone lactylation in a HIFs-dependent manner, thereby transcriptionally activating the expression of platelet-derived growth factor receptor β (PDGFRβ) to promote tumor progress. In turn, overexpression of PDGFRβ positively stimulates histone lactylation [101]. Concerning hepatocellular carcinoma, demethylzeylasteral reduces the lactate level and attenuates histone lactylation, which plays an anti-cancer role by regulating the glycolytic metabolic pathway [102]. In ocular melanoma, elevated histone lactylation effectively promotes the tumorigenesis through up-regulating the transcription of YTHDF2 and further inducing the degradation of PER1 and TP53 mRNAs via binding to their respective m6A sites [103]. Moreover, in colorectal cancer, elevated lactate in tumor-infiltrating myeloid cells induced METTL3 expression by promoting histone lactylation, and further m6A modification on Jak1 mRNA, which promotes its protein translation and strengthened downstream STAT3 signal that enhanced immunosuppressive functions of myeloid cells to promote tumor immune escape.

### Other epigenetic regulations

In addition to those above, lactate-induced lactylation can additionally contribute to DNA repair, embryo implantation, and the improvement of the fatty liver, but it can also lead to the worsening of pulmonary hypertension and proliferative retinopathies [105–110]. Sun Y et al. [105] found that hyperlactylation of PARP1 regulated its ADP-ribosylation activity and might contribute to DNA repair based on an alkynyl-functionalized bioorthogonal chemical reporter, YnLac. The bioorthogonal lactylation chemical reporter opens up new avenues for the functional research and analysis of this newly discovered lactylation in normal physiology and disease. During pregnancy, increased levels of histone H3K18 lactylation and lactate help to maintain glutathione-based redox homeostasis and apoptotic balance, which are essential for successful embryo implantation [106]. However, inhibition of LDHA activity reduces lactate and histone lactylation, thereby impairing embryonic pre-implantation development [107]. Another important benefit is that MPC1 knockout induced lactate accumulation, promoted the lactylation of FASN at the K673 site in hepatocytes to inhibit activity of FASN, and mediated the down-regulation of liver lipid accumulation, as reported by Gao R et al. [108]. On the downside, hypoxia-induced mitochondrial reactive oxygen species (mROS) triggers lactate accumulation and histone lactylation in pulmonary artery smooth muscle cells (PASMCs) by upregulating HIF-1α/PDK1&2/p-PDH-E1α axis, which further promotes the proliferation of PASMCs and vascular remodeling and exacerbates hypoxic pulmonary hypertension [109]. Moreover, hyperlactylation of non-histone YY1 under hypoxia is regulated by p300 as a “writer”. YY1 is directly bound to the promoter of FGF2 and promotes the transcription of FGF2 through its high lactylation, thus promoting the formation of neovascularization. This situation is reversed by the p300/CBP inhibitor A-485 [110].

### Lactylation in different cell biology processes

Increasing studies focus on the role of lactate-mediated lactylation in different cell biology processes to understand the function of protein lactylation. We will specifically discuss lactylation-mediated antitumor immunity and also focus on macrophages, immune cells, and other types of cells.

### Macrophages

Macrophages are a highly heterogeneous cell population and act as scavengers that regulate immune reactions and also participate in the maintenance and restoration of immune homeostasis [111]. Activated macrophages are generally divided into two phenotypes: proinflammatory macrophages, so-called M1-type macrophages, and anti-inflammatory M2-type macrophages [112]. In the early tumor development stage, tumor-associated macrophages facilitate the development of a proinflammatory environment in the tumor, but in later stages, elevated lactate-derived histone H3K18la levels by glycolysis skews macrophage polarization toward the M2 phenotype [14]. In polymicrobial sepsis, lactate-derived HMGB1 in macrophages has elevated lactylation levels, which accumulate in the cytoplasm via exosome secretion and result in endothelial dysfunction [59]. Elevated histone H4K8la levels in macrophages upregulate LINC00152 by reducing the negative regulatory efficiency of YY1 on LINC00152, thereby inhibiting salmonella invasion and inflammatory response and promoting tumor growth [76]. Moreover, TGF-β1 stimulates the increase of lactate production in myofibroblasts and secreted it into the extracellular milieu to promote histone lactylation in macrophages, thereby inducing the expression of some profibrotic mediators [89]. Overall, lactylation affects the metabolic reprogramming and immunomodulatory effects of macrophages. Mainly, it promotes polarization changes that have a positive effect on promoting the repair of damage and tumor phenotype.

### Immune cells

Elevated lactylation of MOESIN at Lys72 in Treg cells mediated by lactate from cancer enhances TGF-β and downstream SMAD3 signaling to regulate the development and function of Treg cells to control tumorigenesis and antitumor therapy [98]. A high level of histone lactylation in tumor-infiltrating myeloid cells induces METTL3 expression and m6A modification of Jak1 mRNA, which leads to the protein translation of Jak1 and the stimulation of downstream STAT3 signal that enhances myeloid immunosuppressive functions [104]. Overall, these studies show that lactylation may have an immunosuppressive effect on the several types of immune cells in tumor microenvironment.

### Neurocytes and osteoblasts

The lactylation level of histone H3 in neural stem cells has been observed to decrease over time during mouse neurogenesis. However, multiple histone Kla levels elevate significantly to orchestrate gene expression changes and widely participate in neuronal differentiation and cell proliferation processes significantly by inhibiting the “eraser” HDAC1-3 or activating the “writer” p300/CBP [63]. Similarly, the increased expression level of H3K18la in osteoblasts promotes the formation of cell mineralized nodules and alkaline phosphatase activity, which plays an important role in the differentiation of osteoblasts [107]. And, the elevated lactylation of LCP1 and histone H3 in neurocytes after cerebral infarction reduce its own degradation and cell viability and enhance the expression of IL-18 and IL-1β and the apoptosis rate of neurocytes [83, 85]. Activated microglia in Alzheimer’s disease are overly lactate and histone lactylated, further promoting glycolytic gene PKM2 expression, resulting in abnormal activation and dysfunction [86]. Moreover, an increase in lactate and histone lactylation levels in neurocytes occurs in response to social defeat stress and stress-associated neural excitation, which are associated with increased anxiety-like behavior [87]. In summary, the existing studies have revealed the non-canonical function of lactylation during nerve and osteogenic differentiation.

#### Tumor cells

The lactylation of c-myc and AK2 in HCC cells affects the degradation and activities of the cells themselves, which result in their viability, migration, and invasion [94, 95]. Similarly, enhanced lactylation of β-catenin in colon cancer cells amplifies the stability and manifestation of β-catenin, thus exacerbating the progression of colon cancer via the Wnt signaling pathway [96]. HCC cells also exhibit elevated lactylation levels of histone H3, which DML can reduce and play an anticancer role by regulating the glycolytic metabolic pathway [102]. Elevated histone lactylation levels have also been reported in lung cancer cells, renal cancer cells, and melanoma cells to promote tumor occurrence and development through mediating HK-1 and IDH3G gene expression, or transcriptionally activating the expression of PDGFRβ and YTHDF2 [100, 101, 103]. Moreover, a prostate cancer cell with elevated lactylation of HIF-1α activates KIAA1199, simulates KIAA1199-mediated angiogenesis and vasculogenic mimicry, and increases depolymerized hyaluronic acid [97].

### Other cell types

Generally, research suggests that lactylation in different cell types functions differently in different conditions. Non-histone YY1 with hyperlactylation regulated by the “writer” p300 in retinal microglia under hypoxia directly interacts with the promoter of FGF2 and promotes its transcription, thereby activating neovascularization [110]. Moreover, lactylation histone H3 is reported to associate with endometrial cells, oocytes, and embryonic cells and contributes to the maintenance of glutathione-based redox homeostasis and apoptotic balance, which are essential for successful embryo implantation [106, 107]. Furthermore, pulmonary hypertension and fatty liver disease are exacerbated by the lactylation change of histone H3 in pulmonary smooth muscle cells and FASN in hepatocytes, respectively [108, 109].

### Lactate/lactylation-targeting drugs

The abundance and immunomodulatory effects of lactate and lactylation may be a novel direction for targeted therapy in various diseases, alone or in combination with other therapeutic strategies. Lactate and its transporter proteins will likely serve as a new therapeutic target, such as targeting MCT1, MCT4, and LDHA, which are currently under preclinical investigations and clinical trials. High LDH levels in the blood and tumor microenvironment are associated with a poor prognosis [113]. The transport capacity of MCT1/4 is critical for intracellular and extracellular lactate levels and transports lactate into and out of the cell according to the concentration of the substrate [114]. Several MCT inhibitors, including syrosingopine, AR-C155858, 7ACC2, BAY8002, SR13800 and AZD3965, have been shown to inhibit MCT activity, but only the MCT1 inhibitor AZD3965 is currently in human clinical trials (NCT01791595) [115–119]. For example, metabolic changes induced by the MCT1 inhibitor AZD3956 (particularly the decrease in lactate export) promote increased infiltration of anti-tumor immune cells (dendritic and natural killer cells), thereby inhibiting tumor growth in mice [120]. Moreover, clinical trials in humans have shown AZD3965 to be well tolerated at doses that deliver target engagement, most commonly with electroretinogram changes, fatigue, and anorexia, all of which are reversible [121]. Co-treatment with anti-PD-1 and the LDHA inhibitor GSK2837808A has a stronger anti-tumor effect than anti-PD-1 therapy alone. Mechanically, lactate degradation reduces regulatory T (Treg) cell induction and tumor growth and enhances anti-tumor immunity [98]. In addition, oxamate and dichloroacetate also inhibit lactate production for the treatment of a variety of tumors as well as metabolic diseases by targeting LDHA and pyruvate dehydrogenase kinase (PDHK), respectively [99, 101]. And, 2-deoxy-D-glucose (2DG), 3-Bromopyruvic acid (3-BrPA), tristetraprolin, and lonidamine have also been reported to be involved in inhibiting hexokinase and thus regulating glycolysis [122–124]. Interestingly, the lactate receptor GPR81 induces chemoresistance in hepatic cancer cells by binding to lactate [125]. And, curcumin and LRH7-G5 can restore the sensitivity of resistant tumor cells to chemotherapy by targeting GPR81 [118, 125]. Overall, targeting lactate and its transporter not only enhances the antitumor responses of the immune system, but also significantly increases their therapeutic efficiency and plays a synergistic effect in combination with checkpoint inhibitors.

Several drugs targeting protein post-translational modifications (i.e., enzymes that catalyze the lactylation and delactylation of proteins) have been shown to be therapeutically effective for a variety of diseases in clinical trials. Highly selective delactylase agonists (e.g., ITSA-1 targeting for HDACs) and inhibitors of lactylation induction (e.g., garcinol targeting for HATs) affect various physiological processes regulated by histone or non-histone lactylation and can be targeted for therapeutic purposes in a variety of diseases [126, 127]. Moreover, the p300/CBP inhibitor A-485 also exerts an anti-retinal neovascularization effect in proliferative retinopathies through the inhibition of lactylation modification of YY1 at the K183 site [110]. Similarily, CPTH6, a selective GCN5 HAT inhibitor, can induce apoptosis in human leukemia cells [128]. Controlling the switch from lactate production and lactylation to acetyl-CoA production and the TCA cycle may provide new opportunities for targeted cancer therapies. Therefore, the exact mechanism of lactylation requires further study to identify novel targets for drug development.

## Conclusions and perspectives

In summary, the new target, protein lactylation, is a “double-edged sword” for human health and diseases. Because it is closely related to multiple physiological and pathological processes, such as neuronal development, embryogenesis, cancer, inflammation, cerebral disease, fibrosis, and so on. Briefly, the positive effects of protein lactylation on human health are typically manifested as a key regulatory role in the differentiated maturity of neural stem cells and osteoblast differentiation and bone formation, as well as transcription elongation of embryo cleavage-related genes. In addition, lactylation also contributes to DNA repair, embryo implantation, and the improvement of the fatty liver. However, on the other hand, elevated lactylation may have either a causative or predisposing role in the worsening of cancer, inflammation, fibrosis, and brain diseases, pulmonary hypertension, and even proliferative retinopathies. These perspectives are critically significant and are first described in detail in this review. Although the functions of protein lactylation in health and disease have been reported, to aid in the development of more targeted lactylation inhibitors/agonists and facilitate their application in clinical practice, additional studies exploring the concrete molecular mechanisms of lactylation are needed.

## Data Availability

All data generated or analyzed during this study are included in this published article.
